# Characteristics and Clinical Relevance of Candiduria in Hospitalized Patients

**DOI:** 10.3390/jcm15051881

**Published:** 2026-03-01

**Authors:** Leticia Castellano-Sánchez, Antonio Rosales-Castillo, María del Carmen Olvera-Porcel, José Gutiérrez-Fernández

**Affiliations:** 1 Microbiology Laboratory, Hospital Universitario Virgen de las Nieves, Instituto de Investigación Biosanitaria de Granada, 18014 Granada, Spain; leticia.castellano.sspa@juntadeandalucia.es; 2 Clinical Medicine and Public Health Doctoral Program, Graduate School, University of Granada, 18014 Granada, Spain; 3 Department of Internal Medicine, Hospital Universitario Virgen de las Nieves, Instituto de Investigación Biosanitaria de Granada, 18014 Granada, Spain; 4 Biostatistics Unit, Hospital Universitario Virgen de las Nieves, Instituto de Investigación Biosanitaria de Granada, 18014 Granada, Spain; mariac.olvera.sspa@juntadeandalucia.es

**Keywords:** *Candida*, urine culture, *Nakaseomyces glabratus*, risk factors, antifungal agents

## Abstract

**Background:** Candiduria is a common finding in hospitalized patients, particularly in intensive care units (ICUs) and in those with indwelling urinary catheters. Additionally, *Candida* spp. is among the most frequent causes of healthcare-associated urinary tract infections and can lead to severe clinical manifestations in specific scenarios involving risk factors. **Objective:** The objective of this study is to describe and analyze the epidemiological features, clinical risk factors, therapeutic approaches, and clinical outcomes in a cohort of hospitalized patients with candiduria at a regional hospital. **Methods:** This was a retrospective, descriptive, cross-sectional study based on the selection of 207 urine cultures positive for *Candida* spp. between 1 February 2024, and 31 August 2024, at the Microbiology Laboratory of the Virgen de las Nieves University Hospital in Granada, Spain. **Results:** The most frequently isolated species was *Nakaseomyces glabratus* (42.03%), with no sex differences and a predominant occurrence in ICU patients (36.71%). Most patients had comorbidities (86.47%), urinary catheters (72.46%), and prior antibiotic use (75.85%). Less than half of the cases presented clinical symptoms (41.55%). Antifungal therapy was administered in 38.65% of cases, predominantly fluconazole (61/207; 29.47%), followed by echinocandins (12/207; 5.8%). Use of sodium-glucose co-transporter-2 inhibitors (SGLT2i) was documented in 36.71% of patients. **Conclusions:** Candiduria is more frequently observed in elderly patients with multiple comorbidities, ICU admission, indwelling urinary catheters, prior antibiotic therapy, and SGLT2i use. Fewer than half of the episodes are symptomatic. Non-*albicans* yeast species, which often display distinct resistance patterns, are increasingly prevalent, with *N. glabratus* being the most frequently isolated.

## 1. Introduction

Clinically relevant opportunistic yeasts comprise a broad and heterogeneous group of species, including members of the genus *Candida* as well as other related genera, many of which are part of the human microbiome. Nevertheless, *Candida albicans* remains the predominant species.

*Candida* can cause a wide spectrum of clinical conditions [1], ranging from cutaneous (e.g., intertrigo, folliculitis), gastrointestinal (e.g., esophagitis, peritonitis), and genitourinary infections (e.g., cystitis/pyelonephritis, vulvovaginitis, balanoposthitis), to candidemia and disseminated infections, including endocarditis [2].

Candiduria is a frequent finding among hospitalized patients, particularly in intensive care units (ICUs); a prospective multicenter study found an incidence of candiduria of 22% in patients in intensive care units with a stay longer than 7 days [3] and Altinbas et al. reported that up to 67.3% of cases of funguria originated from Intensive Care Units [4]. The main challenge lies in differentiating colonization from true infection, as a significant proportion of cases represent asymptomatic colonization. Nevertheless, yeast-like fungi is considered the second most frequent cause of healthcare-associated urinary tract infections (HA-UTIs) [5]. In a previous study by Bouza et al. [6], *Candida* species were estimated to be responsible for up to 9.4% of HA-UTIs. Furthermore, *Candida* spp. has been identified in up to 18% of UTIs in patients with urinary catheters and in approximately 1% of UTIs in critically ill patients [7]. In other populations, such as patients with spinal cord injury, the prevalence of candiduria was 17% in one study, with antibiotic use and catheterization being the two most important factors [8].

After *C. albicans*, the most commonly isolated species in most studies are *Nakaseomyces glabratus* and *Candida tropicalis* [9]. However, an increasing prevalence of these non-*albicans Candida* species has been reported, in some cases even surpassing *C. albicans* [5]. Recognizing non-*albicans Candida* species is clinically important, as they often exhibit different antifungal resistance profiles and tend to be more prevalent in specific clinical contexts, such as in diabetic patients [10,11].

Identified risk factors for candiduria include the presence of urinary catheters or nephrostomy tubes, urinary tract abnormalities, abdominal surgery, broad-spectrum antibiotic use, female sex, age over 65, diabetes mellitus, immunosuppression, mechanical ventilation, and ICU stay [3]. It should be noted that most patients with candiduria are asymptomatic and current laboratory techniques do not allow differentiation between colonization and infection [12]; nevertheless, it may be the only evidence of invasive candidiasis in some patients [13].

The fact that most cases of candiduria are asymptomatic and are associated with potentially modifiable risk factors (such as the presence of an indwelling urinary catheter) makes their therapeutic approach challenging. Several management algorithms have been proposed to improve and standardize care, including the IDSA guidelines [14] and the Fisher classification [15]. Both emphasize the importance of controlling predisposing risk factors and reserving antifungal treatment for specific clinical situations.

As previously described, the difficulty in distinguishing colonization from true infection, as well as determining whether antifungal treatment is warranted, are two key challenges in the management of candiduria. The objective of this study is to describe and analyze the epidemiological characteristics, clinical risk factors, therapeutic approaches, and clinical outcomes of a series of hospitalized patients with candiduria at a regional hospital.

## 2. Materials and Methods

A cross-sectional, descriptive, and retrospective study was conducted using data extracted from the laboratory information system (LIS) Modulab^®^ (Werfen Laboratories, Barcelona, Spain). A list of all urine cultures corresponding to clinical episodes processed between 1 February 2024, and 31 August 2024, by the Microbiology Laboratory of the Virgen de las Nieves University Hospital (Granada, Spain) was obtained, in which *Candida* spp. were isolated. A total of 207 urine culture reports were selected, and the corresponding patients were identified for the review of clinical variables through the electronic medical records. The selected study timeframe was defined to include the most recent and complete set of microbiologically confirmed cases available in the laboratory information system at the time of data extraction, allowing for the inclusion of a sufficient number of independent clinical episodes while ensuring homogeneity in diagnostic procedures and laboratory methodologies. The main limitations of the study are its observational and single-center design, as well as the inability to establish temporal or causal relationships.

Samples were obtained from patients attended in the emergency departments, medical and surgical hospitalization wards, and the intensive care unit, which provide healthcare coverage to the population of the city of Granada and the municipalities within its metropolitan area of influence.

All episodes included were distinct, occurring at least six weeks apart from any previous episode, when applicable. The only exclusion criterion was the duplication or repeated microbiological testing of the same clinical episode.

Urine samples were collected under conditions minimizing contamination, and in the context of suspected urinary tract infection (UTI), using midstream clean-catch, permanent catheter, temporary catheterization, pediatric urine collection bags, or nephrostomy catheter. Urine sample analysis was performed in accordance with the procedures established in the European guidelines [16]. In our study, leukocyturia is defined as the presence of ≥5 leukocytes per high-power field (400X), and erythrocyturia as the presence of ≥5 erythrocytes per high-power field (400X).

For the purposes of the study, candiduria was defined as the isolation of one or more yeast microorganisms in a urine culture corresponding to an independent clinical episode, according to the type of specimen. Urine culture detection and interpretation were carried out using chromogenic media (CHROMID^®^ CPS^®^ and CHROMID^®^ Candida, bioMérieux, Craponne, France) after 24–48 h of incubation at 37 °C. Analyses were conducted of results obtained from the culture of isolates detected in these samples, which were processed according to the standard protocol of the laboratory [17]. In samples obtained by spontaneous micturition from permanent probe, collector bag, or nephrostomy catheter, a significant result was defined by a count ≥10^5^ CFU/mL or by a count of ≥10^4^ CFU/mL for a single microorganism in the presence of >40 leukocytes/mL in uncentrifuged urine; urine cultures with a growth of >2 microorganisms were considered contaminated. In samples obtained by provisional transurethral catheterization, a significant result was defined by a count ≥10^4^ CFU/mL of one or two microorganisms. MALDI Biotyper (Bruker Daltonics, Billerica, MA, USA) was used to identify the microorganisms grown in culture. These criteria were uniformly applied to all episodes included in the study [18].

Antifungal susceptibility testing (AST) was performed for all clinical isolates. Minimum inhibitory concentrations (MICs) were determined using the Sensititre™ YeastOne system (Thermo Fisher Scientific, Waltham, MA, USA) according to the manufacturer’s instructions. MICs were interpreted using current EUCAST clinical breakpoints when available; where EUCAST breakpoints were not available, epidemiological cutoff values (ECVs) were applied. Quality-control strains were included and processed in parallel.

Clinical and epidemiological variables were obtained from the electronic medical records and included: sex, age, yeast species, type of sample (midstream, catheter-obtained, or permanent catheter), presence of urinary catheter, nephrostomy, or nephrourological anatomical abnormalities, medical comorbidities (hypertension, chronic obstructive pulmonary disease, diabetes mellitus, chronic kidney disease), length of hospital stay, ICU admission, mechanical ventilation, parenteral nutrition, abdominal surgery, presence of sepsis/septic shock, pneumonia, central venous catheter, presence of immunosuppression (solid organ transplant, active neoplasia or chemotherapy, liver cirrhosis, chronic corticosteroid therapy, hemodialysis, or HIV infection), associated symptoms (dysuria, fever, and/or hematuria in the absence of any other associated cause), isolation in other sample types, urine analysis findings, antifungal treatment, follow-up urine culture, and clinical outcome.

### 2.1. Statistical Analysis

A descriptive statistical analysis was performed for all variables. Measures of central tendency and dispersion were calculated for continuous variables, and absolute and relative frequencies were reported for categorical variables.

To compare quantitative data across candiduria groups, the Kruskal–Wallis test was used. Categorical variables were compared using the chi-squared test or Fisher’s exact test, as appropriate. Statistical analyses were performed using Stata version 16.1.

### 2.2. Ethical Considerations

Ethical approval for this retrospective study was obtained from the Institutional Review Board of Hospital Virgen de las Nieves de Granada, España (approval number: SICEIA-2024-001491). Given the observational design and the use of anonymised routinely collected data, the requirement for informed consent was waived in accordance with national regulations and the Declaration of Helsinki.

## 3. Results

The general descriptive characteristics of the included cases are summarized in Table 1. The clinical departments from which the samples originated were, in descending order of frequency: Intensive Care Unit (36.71%; 76/207), Emergency Department (30.43%; 63/207), Internal Medicine (15.5%; 32/207), Surgical Services (9.2%; 19/207), Gynecology (2.9%; 6/207), and other departments (5.3%; 11/207).

Among conditions associated with immunosuppression, which were present in 30.9% of cases, the following were observed: chronic corticosteroid use (12/207; 5.8%), neutropenia (3/207; 1.45%), solid malignancy (24/207; 11.59%), active chemotherapy (8/207; 3.86%), liver cirrhosis (7/207; 3.38%), solid organ transplantation (5/207; 2.42%), HIV infection (3/207; 1.45%), and hemodialysis (2/207; 0.97%). Three cases of neutropenia were identified. In all three cases, *N. glabratus* was isolated and candidemia was present. Two patients had profound neutropenia (absolute neutrophil count <100/mm^3^), and one had an absolute neutrophil count <500/mm^3^. All three patients died from other complications without recovery of neutrophil counts due to their underlying conditions (acute myeloblastic leukemia in two cases and metastatic lung cancer in the remaining case).

Concomitant isolation of *Candida* spp. from other clinical specimens occurred in 9/207 (4.35%) of cases, primarily from blood cultures (1.93%) and bronchial aspirates (1.93%). In most cases (7/9), the same species was identified (*N. glabratus* [n = 5] and *C. albicans* [n = 2]). In the remaining two cases, *Pichia kudriavzevii* and *N. glabratus* were isolated from endocervical samples, while *C. albicans* was isolated in urine cultures. Co-isolation with other microorganisms was observed in 4.83% (10/207) of the episodes. The microorganisms most frequently co-identified were *Escherichia coli* (5/10), *Enterococcus faecalis* (3/10), and *Enterococcus faecium* (2/10).

The *Candida* species identified, in order of frequency, were: *N. glabratus* (87/207; 42.03%), *C. albicans* (69/207; 33.33%), *C. tropicalis* (34/207; 16.43%), *Candida parapsilosis* (7/207; 3.38%), *P. kudriavzevii* (6/207; 2.9%), and *Clavispora lusitaniae* (4/207; 1.93%). All *C. albicans* isolates were susceptible to fluconazole. Among *N. glabratus* isolates (n = 87), five isolates (5/87; 5.7%) were resistant to fluconazole and the remaining isolates were classified as susceptible. No resistance to echinocandins was detected in the collection.

Regarding clinical presentation, 41.55% of episodes (87/207) showed associated symptoms, mainly dysuria (54/207; 26.09%), fever (22/207; 10.63%), and hematuria (19/207; 9.18%) in the absence of another infectious or alternative cause. Urinalysis was performed in 74.4% of cases (154/207), with common findings including leukocyturia (145/154; 94.16%), erythrocyturia (11/154; 71.42%), and glycosuria (90/154; 58.44%). The presence of a fungal ball was not documented in any case; however, various anatomical abnormalities of the urinary tract were identified in 3.86% of cases, including ureteral stenosis, megaureter, and ureteropelvic junction stenosis.

In terms of therapeutic management, urinary catheters were removed in 22.22% of cases (46/207) and replaced in 19.81% (41/207). Candiduria resolved in fewer than seven days in 30/46 (65.2%) cases following catheter removal, and in 22/41 (53.7%) cases after catheter replacement, without the need for antifungal treatment. Antibiotic therapy was maintained in nearly all cases where it had been initiated (150/155; 96.8%). Antifungal treatment was administered in 38.65% of patients (80/207), primarily fluconazole (61/207; 29.47%), followed by echinocandins (12/207; 5.8%) and amphotericin B (7/207; 3.38%). In the fluconazole-treated group, the isolated species were, in order of frequency: *C. albicans* (22/61), *N. glabratus* (18/61), *C. tropicalis* (19/61), *C. parapsilosis* (1/61), and *P. kudriavzevii* (1/61). 65.6% (40/61) achieved negative urine cultures, and no associated deaths were recorded. In the echinocandin-treated group, the species distribution was: *C. albicans* (6/12), *N. glabratus* (5/12), and *C. parapsilosis* (1/12). 58.3% (7/12) achieved negative urine cultures, and one directly related death was recorded (patient with acute leukemia and associated candidemia due to *N. glabratus*). Finally, in the amphotericin B group, the species were *N. glabratus* (3/7; 42.9%) and *C. parapsilosis* (4/7; 57.1%). 28.6% (2/7) had a negative follow-up urine culture, and no directly associated deaths were recorded. Among the untreated patients (n = 127), 53.5% (68/127) had a negative follow-up urine culture, and deaths were recorded in this group.

As previously mentioned, only five cases of fluconazole resistance were identified among *N. glabratus* isolates; all *C. albicans* isolates tested were susceptible. Of these five resistant cases, two patients received fluconazole (likely prior to availability of antifungal susceptibility testing results), whereas no antifungal therapy was administered in the remaining three cases. No deaths were recorded, and follow-up urine cultures were negative in 3/5 cases (60%).

Follow-up urine cultures were performed in 79.22% of patients (165/207), and 72.1% of these (119/165) were negative. Two deaths (0.97%) were directly related to the candiduria episode (both with associated candidemia), while thirty-two patients (15.46%) died due to other causes.

A comparative analysis of the three most frequent species (*C. albicans, N. glabratus,* and *C. tropicalis*) is detailed in Table 2. Statistically significant differences were observed across several variables:

- *N. glabratus* was associated with older age (median 80 years), diabetes mellitus, mechanical ventilation, and SGLT2 inhibitor use.
- *C. albicans* was associated with mechanical ventilation, parenteral nutrition, pneumonia, and central venous catheter use.
- *C. tropicalis* was associated with prior antibiotic use (within the last 3 months), sepsis/septic shock, positive follow-up urine culture, fluconazole administration, and longer hospital stay.

## 4. Discussion

This study analyzed and reviewed the clinical characteristics of candiduria episodes in hospitalized patients, including associated risk factors, clinical course, and therapeutic management. The growing importance of *Candida* spp. lies in its etiological role in healthcare-associated urinary tract infections (UTIs), as previous reports have estimated that *Candida* spp. may account for 10–15% of UTIs in hospitalized patients and up to 18% in those with indwelling urinary catheters [6,19,20]. In specific settings such as Intensive Care Units (ICUs), *Candida* spp. may be responsible for up to 25% of UTIs [21], with length of stay being a major contributing factor [22]. One study found that up to 22% of patients with ICU stays exceeding one week developed candiduria [23].

From a microbiological standpoint, several factors may lead to underestimation of yeast species, especially non-*albicans* species, due to challenges in their identification. These include slower growth (sometimes beyond 48 h) of non-*albicans* species on chromogenic media [24], reduced sensitivity of standard urine cultures, and the small colony size of *N. glabratus*, which hampers detection by cytometry [25].

Among the risk factors analyzed, the presence of a urinary catheter is particularly noteworthy. *C. albicans* is the second most common cause of catheter-associated UTIs, following Escherichia coli. Bouza et al. [6] reported that 16.6% of catheter-associated healthcare-related UTIs were caused by *Candida* spp., while in the study by Padawer et al. [26], this figure reached 19.49%. In our cohort, the prevalence of urinary catheter use was high (72.46%), with no significant difference across *Candida* species. Notably, removal of the catheter alone can lead to resolution of candiduria in more than 40% of patients, and replacement in up to 20% [27]. In our sample, more than 40% underwent catheter removal or replacement as part of therapeutic management.

Other well-established risk factors for candiduria include urinary tract abnormalities, abdominal surgery, broad-spectrum antibiotic use, female sex, age over 65, diabetes mellitus, immunosuppression, mechanical ventilation, and ICU admission. Our findings are consistent with these data: median age was 72 years, more than one-third (36.71%) were admitted to the ICU, over half (58.45%) had diabetes, and three-quarters had received antibiotics previously or were on active therapy. Additionally, nearly one-third of patients had some form of immunosuppression, primarily active malignancy. Notably, the continuation of antibiotic therapy despite *Candida* isolation was observed in 96.8% of cases—while this may be appropriate when bacterial coinfection is suspected, unnecessary antibiotic use is a known risk factor for fungal overgrowth.

Although candiduria is often asymptomatic—particularly in cases of colonization—our study found a relatively high proportion of symptomatic episodes (over 40%), with fever and dysuria being the most common. It is worth noting that a proportion of patients with candidemia may initially present with candiduria, making close clinical monitoring essential—especially in critically ill or ICU patients [28,29].

We also highlight the increasing prevalence of non-*albicans* yeast species. In our study, the most frequently isolated species was *N. glabratus*, differing from earlier findings, including our own previous research in 2016, where *C. albicans* was predominant [18]. The time elapsed between the two studies likely reflects a shift in species distribution. In fact, studies such as that by Richards et al. [5] report non-*albicans* species in more than 50% of isolates, with fluconazole use identified as a major risk factor—particularly for *N. glabratus* selection [30]. This has significant clinical implications given the inherently higher resistance of *N. glabratus* to commonly used antifungal agents [31].

Another notable finding was the high frequency of sodium-glucose cotransporter-2 inhibitor (SGLT2i) use (36.71%) in our population. These agents promote glycosuria as part of their mechanism of action and may create a favorable environment for fungal growth. There are increasing reports linking SGLT2i use to candiduria and even candidemia [32,33].

Given the complexity in deciding when antifungal treatment is appropriate, several clinical algorithms have been proposed. Fisher et al. [15] proposed a five-category model: (1) asymptomatic candiduria in healthy patients, (2) asymptomatic candiduria in outpatients with risk factors, (3) asymptomatic candiduria in hospitalized patients with risk factors, (4) symptomatic candiduria (cystitis, pyelonephritis, prostatitis, orchiepididymitis, fungal ball), and (5) candiduria in unstable patients. Antifungal therapy is recommended in cases of renal infection, neutropenia, hemodynamic instability, or disseminated candidiasis. Persistent candiduria despite adequate treatment should prompt investigation for local complications requiring surgical intervention (e.g., fungal ball, abscess, anatomical abnormalities).

The IDSA guidelines [14] categorize candiduria based on symptomatology. In symptomatic cases, antifungal treatment is indicated based on clinical presentation (e.g., cystitis, pyelonephritis, fungal ball, epididymo-orchitis, prostatitis). In asymptomatic cases, clinical monitoring and removal of risk factors are preferred; antifungal therapy is only advised in high-risk scenarios (e.g., neutropenia, low birth weight neonates).

Regarding treatment, fluconazole remains the drug of choice due to its high oral bioavailability and urinary excretion. Echinocandins, although useful in certain systemic fungal infections, have limited renal excretion, though favorable outcomes have been reported [34]. In our cohort, echinocandins were used in 12 cases (5.8%) with one death (due to *N. glabratus*), while no deaths occurred in the fluconazole-treated group. General treatment regimens include:

- For cystitis/pyelonephritis: fluconazole 200–400 mg/day for 14 days; alternatives include flucytosine 25 mg/kg every 6 h for 7 days, or amphotericin B deoxycholate bladder irrigation (50 mg/L) for 5 days.
- For prostatitis and epididymo-orchitis: fluconazole 400 mg/day orally for 4 weeks, with surgical drainage if needed.
- For fungal balls: similar systemic therapy plus mandatory surgical drainage.

This study has several limitations, including its observational and single-center design, and the absence of a control group. However, its strengths lie in the large sample size, comprehensive collection of clinical variables, and species-specific analysis of the three most commonly isolated *Candida* species.

## 5. Conclusions

Candiduria is more frequently observed in elderly patients with multiple comorbidities, ICU admission, indwelling urinary catheters and prior antibiotic therapy. SGLT2 inhibitor use was common (over one-third), and fewer than half of the patients presented with clinical symptoms. In our study, the most frequently identified species was *N. glabratus*, followed by *C. albicans* and *C. tropicalis*. Antifungal treatment was administered in more than one-third of cases, most frequently with fluconazole, and less commonly with echinocandins.

## Figures and Tables

**Table 1:** General clinical and epidemiological characteristics.

Variables	
Median age (IQR)	72 (19–95)
Male sex, n (%)	104 (50.24)
Hospital stay, days (IQR)	10.7 (0–90)
ICU stay n (%)	76 (36.71)
ICU stay, days (IQR)	19 (7–100)
Hypertension, n (%)	179 (86.47)
COPD n (%)	52 (25.12)
Chronic kidney disease, n (%)	88 (42.51)
Diabetes mellitus, n (%)	121 (58.45)
iSGLT2, n(%)	76 (36.71)
Previous episodes of candiduria, n (%)	14 (6.76)
Urinary catheter, n (%)	150 (72.46)
Concomitant antibiotic treatment, n (%)	155 (74.88)
Antibiotic use in previous 3 months, n (%)	157 (75.85)
Immunosuppression, n (%)	64 (30.9)
Urinary tract anatomical abnormalities, n (%)	8 (3.86)
Invasive mechanical ventilation, n (%)	35 (16.91%)
Parenteral nutrition, n (%)	29 (14.01%)
Pneumonia, n (%)	21 (10.14%)
Sepsis/septic shock, n (%)	43 (20.77%)
Previous abdominal surgery, n (%)	13 (6.28%)
Central venous catheter, n (%)	57 (27.54%)

iSGLT2: sodium-glucose cotransporter 2 inhibitors; COPD: chronic obstructive pulmonary disease; ICU: Intensive Care Unit.

**Table 2:** Clinical and epidemiological characteristics of different *Candida* species isolated.

	*C. albicans* (69)	*N. glabratus* (87)	*C. tropicalis* (34)	*p* Value
Age Median (IQR)	71 (25)	80 (16)	70 (12)	0.010
Male sex n (%)	39 (59.62)	39 (44.83)	19 (55.88)	0.288
Diabetes Mellitus n (%)	35 (50.72)	63 (72.41)	14 (41.18)	0.002
ICU stay n (%)	28 (40.58)	14 (16.09)	12 (35.29)	0.002
Urinary catheter n (%)	52 (75.36)	59 (67.82)	27 (79.41)	0.357
Antibiotic n (%)	53 (76.81)	62 (71.26)	26 (76.47)	0.694
Antibiotic previous 3 months n %)	46 (66.67)	69 (79.31)	32 (94.12)	0.006
Mechanical ventilation n (%)	21 (30.43)	11 (30.43)	0 (0.00)	0.000
Parenteral nutrition n (%)	14 (20.29)	10 (11.49)	0 (0.00)	0.013
Pneumonia n (%)	11 (15.94)	9 (10.34)	0 (0.00)	0.046
Sepsis/shock n (%)	12 (17.39)	13 (14.94)	12 (35.29)	0.034
Central venous catheter n (%)	26 (37.68)	14 (16.09)	10 (29.41)	0.009
Positive control urine culture n %)	7 (13.21)	15 (24.62)	15 (50.00)	0.001
Associated symptoms n (%)	23 (33.3)	43 (49.40)	14 (41.20)	0.129
iSGLT2 n (%)	17 (24.60)	47 (54.00)	7 (20.60)	0.000
Fluconazole treatment n (%)	22 (31.90)	18 (20.70)	19 (55.90)	0.001
Length of hospital stay (median- IQR)	10 (18)	3 (10)	11.5 (14)	0.002

iSGLT2: sodium-glucose cotransporter 2 inhibitors.

## Data Availability

The original contributions presented in this study are included in the article. Further inquiries can be directed to the corresponding author.
